# Glyco-Architectural Remodelling of the Feline Heart: Age- and HCM-Related Insights from Lectin Histochemistry

**DOI:** 10.3390/life16010020

**Published:** 2025-12-22

**Authors:** Irina Constantin, Romelia Pop, Andrada Negoescu, Dragoș Hodor, Mara Georgiana Haralambie, Raluca Marica, Flaviu-Alexandru Tăbăran

**Affiliations:** Department of Pathology, University of Agricultural Sciences and Veterinary Medicine, 400372 Cluj-Napoca, Romania; irina.constantin@usamvcluj.ro (I.C.); andrada.negoescu@usamvcluj.ro (A.N.); dragos.hodor@usamvcluj.ro (D.H.); mara-georgiana.haralambie@usamvcluj.ro (M.G.H.); raluca.marica@usamvcluj.ro (R.M.); alexandru.tabaran@usamvcluj.ro (F.-A.T.)

**Keywords:** cats, confocal microscopy, heart, histochemistry, hypertrophic cardiomyopathy, lectin, tissue microarray

## Abstract

Glycosylation plays a critical role in maintaining cardiac structure and function, yet its modulation during aging and hypertrophic cardiomyopathy (HCM) in feline hearts remains uncharacterized. This study provides a systematic analysis of lectin-binding patterns in feline myocardium across different age groups and disease states. Post-mortem feline hearts (*n* = 64), classified by age (newborn to senior) and diagnostic status (healthy vs. HCM-affected), were evaluated using tissue microarrays stained with five plant-derived lectins—Concanavalin A (ConA), Wheat Germ Agglutinin (WGA), RCA (*Ricinus communis* Agglutinin I), Tomato (*Lycopersicon esculentum* Agglutinin), and *Griffonia (Bandeiraea) simplicifolia* Lectin I (BS)—alongside Draq5 nuclear counterstaining. Lectin histochemistry revealed distinct, region-specific glycosylation patterns, with notable remodelling in both aged and HCM-affected hearts. These glycan alterations reflect underlying molecular and structural changes associated with cardiac aging and pathology. Although lectin histochemistry has been used to examine cardiac glycosylation in species such as mice, rats, zebrafish, and humans, comparable data for felines have been lacking, even if domestic cat represents a spontaneous model for human HCM. This study provides the first essential step in characterizing the feline cardiac glycosylation. The observed shifts in lectin-binding profiles reveal specific remodelling associated with aging and HCM in cats. These results provide a foundation for future studies assessing the utility of glycan motifs as potential post-mortem markers of disease progression in felines.

## 1. Introduction

Animal cell surfaces contain protein and lipid glycoconjugates, which serve diverse and essential biological functions [1]. Glycoconjugates show a heterogeneous distribution across tissues and species [2,3]. The sugar moieties of these protein- and lipid-linked glycoconjugates exhibit specific binding activity with lectins [4]. Lectins, a major protein family [2,3], are widely used in histochemical applications, particularly plant-based lectins, which can map glycoconjugate patterns in cells and tissues [5,6,7]. Lectin histochemistry is used to identify residual sugars on cell membranes and within intracellular organelles, revealing the different patterns of glycoconjugates expression in tissues [1,8,9,10,11]. In comparison with immunohistochemistry, a laboratory technique in which antibodies are used to detect antigens in cells within a tissue section [12,13,14,15,16], lectin histochemistry can offer a complementary perspective in cardiac studies [17,18,19,20,21,22].

As a complex organ, the mammalian heart is prone to various age-related structural and functional alterations that may contribute to the development of heart disease and heart failure. Among these alterations, increased fibrosis, enlargement of cardiomyocytes, and reduced autophagy activity within the cells, are included [23,24,25,26,27]. These degenerative processes are accompanied by a range of biochemical and molecular alterations, including elevated oxidative stress, disrupted energy metabolism, and remodelling of collagen and other extracellular matrix components [23,28,29,30,31].

The HCM is the most common cardiac disease in cats, affecting an estimated 10–15% of the domestic cat population. While it can occur in cats of any breed, age, or sex, certain breeds such as Maine Coons and Ragdolls are more predisposed due to known genetic factors [32,33,34]. Feline HCM closely resembles the human form of the disease, making it a valuable large animal model that is both genotypically and phenotypically relevant to human HCM [33,34].

Glycosylation plays a vital role in the cardiovascular system, influencing protein stability, cell–cell communication, and extracellular matrix organization [35]. Evidence shows that aging hearts display distinct alterations in glycosylation profiles, which may contribute to structural remodelling and functional decline [36]. Furthermore, HCM involves metabolic dysregulation, including impaired glucose oxidation and shifts in substrate utilisation, which may in turn affect glycan biosynthesis and remodelling of cardiac glycoconjugates [37].

Although lectin histochemistry has been applied to cardiac tissue in various species, such as mice, rats, zebrafish, and even humans, to assess glycosylation changes during development or disease, these findings cannot be directly extrapolated to felines [15,17,38,39,40,41,42]. Glycan expression exhibits species- and tissue-specific variability, and the domestic cat, with its spontaneous and genetically relevant model of HCM, represents an underexplored area in the current literature [33,34]. To the authors’ best knowledge, to date, lectin-binding patterns in feline cardiac tissue during aging or HCM have not been systematically described.

The aim of this study was to assess whether aging and disease are associated with detectable changes in the glycol architecture of the feline heart, using lectin histochemistry as a descriptive tool. Specifically, the binding patterns of five plant-derived lectins, selected based on their glycan specificity and relevance to cardiac biology, were investigated: ConA binds α-mannose and α-glucose residues, abundant in high-mannose N-glycans involved in glycoprotein folding and intracellular trafficking within cardiomyocytes. Its signal declines with age and disease, reflecting impaired protein processing and extracellular matrix remodelling [39,43,44]. The Wheat Germ Agglutinin WGA recognizes N-acetylglucosamine (GlcNAc) and sialic acid residues that are critical for sarcolemmal glycoprotein structure, electrophysiological stability, and stress responses. The WGA is often used as a marker of cardiomyocyte membrane integrity and fibrosis [22,40,45,46]. The RCA lectin detects terminal β-D-galactose residues, which can be masked by sialic acids. The RCA binding reflects developmental and pathological alterations in galactosylation and may signal shifts in glycan branching or metabolic dysfunction [38,39,47,48]. The Tomato lectin binds poly-N-acetylglucosamine (poly-GlcNAc) sequences frequently present on extracellular matrix components and cell adhesion molecules. Alterations in its binding pattern are associated with aging and fibrosis [17,39,41]. The plant BS lectin BS targets terminal α-galactosyl and N-acetylgalactosamine residues. It is sensitive to vascular remodelling, endothelial activation, and developmental changes in glycoprotein expression [17,39,42].

## 2. Materials and Methods

### 2.1. Sample Collection, Histological Processing, and Tissue Microarray Preparation

This study was conducted on a total of 64 feline hearts collected during necropsy examinations of spontaneous cases from both male and female subjects, ranging from newborn to 24 years old, at the Pathology Department of University of Agricultural Sciences and Veterinary Medicine, Cluj-Napoca, Romania. The necropsies and histological analysis of the subjects took place between October 2021 and December 2024.

The necropsy examinations were performed following a previously described technique focused on heart pathology, to preserve the heart structure during the examination [49]. During the evaluation, the hearts were inspected, harvested, weighed, followed by the preservation of the samples in 10% neutral buffered formalin (NBF) for at least 48 h until complete fixation. The formalin fixed hearts were further examined by four-chamber dissection technique [49]. The samples were routinely processed, and paraffin embedded tissues were sectioned at a 2 to 4 μm thickness and stained with hematoxylin and eosin stain (H&E stain) [50].

The control group consisted of hearts showing normal gross morphology and normal histological features, with no evidence of cardiomyopathy or other cardiac pathology. Control samples met the same preservation criteria as HCM samples and were excluded if autolysis, tissue damage, or any cardiac abnormalities were present. In addition to hearts with normal gross and microscopic morphology, only those showing clear pathological evidence of HCM were selected for analysis. The inclusion criteria for HCM were based on established gross, histological, and morphometric characteristics [51,52,53]. Specifically, hearts weighing more than 18 g were considered for cardiomegaly and selected for further examination [32,54,55]. Grossly, HCM was identified by concentric left ventricular hypertrophy, often with narrowing of the ventricular lumen. Histological confirmation required the presence of characteristic features such as cardiomyocyte hypertrophy with nuclear enlargement, interstitial or replacement fibrosis, and myofiber disarray [51,52,53].

Hearts were excluded from the study if they presented findings suggestive of other cardiac conditions such as dilated cardiomyopathy, restrictive cardiomyopathy, myocarditis, or cardiac neoplasia. Additionally, autolyzed or incompletely preserved samples that precluded reliable pathological assessment were excluded.

During necropsy and histopathological evaluation, cases were screened for comorbidities. Cats with histological or gross evidence of concurrent systemic disease, including chronic kidney disease, hyperthyroidism, systemic neoplasia, myocarditis, or advanced inflammatory/infectious lesions, were excluded from the study to minimize confounding effects on cardiac glycosylation.

Both male and female cats were included, and sex was recorded for all individuals. While sex differences were not used as stratifying criteria due to sample size limitations, distribution across groups was approximately balanced, and no selection bias was introduced. Future studies with larger cohorts could further examine the impact of sex on cardiac glycosylation in HCM.

In the present study, all age datasets were categorized as Kitten (until 1 year), Young Adult (1–6 years), Mature Adult (7–10 years), and Senior (>10 years) for data consistency, using the feline life stages described in an inferred guide [56]. Each group was further subdivided into control and HCM categories based on necropsy and histological examinations (Table 1).

For a facile process, a 3D printer was used to create a tissue microarray (TMA) mold. Melted silicone was poured into the 3D-printed mold and allowed to set for 12 h to form the final TMA mold. Using this silicone mold, paraffin blocks were prepared, resulting in tissue microarrays with 12 separate tissue cores.

Biopsy punches measuring 5 mm in diameter were systematically used to obtain myocardial samples from three predefined anatomical regions in all cases: left ventricle (LV), right ventricle (RV), and left atrium (AT). To ensure consistency and minimize regional variability, ventricular samples were always collected from the free walls of the LV and RV, precisely 5 mm below the atrioventricular junctions, while atrial samples were taken from the free atrial wall, 2 mm above the atrioventricular junctions. This standardized approach yielded 192 biopsies in total (64 per region). Following collection, samples were deparaffinized, placed in predetermined cores of TMAs, and embedded in warm paraffin to ensure uniform processing. Completed TMA blocks were cooled on a histology hob, and serial sections of 4 μm thickness were prepared for subsequent histochemical analyses. To enhance reproducibility and reliability, downstream statistical analyses were performed separately for each anatomical region, and adjustments for multiple comparisons were applied where appropriate.

### 2.2. Chemical and Reagents

The kits containing Concanavalin A, Wheat Germ Agglutinin and *Ricinus communis* Agglutinin (Rhodamine Lectin Kit I catalog no. RLK-2200), and *Lycopersicon esculentum* Lectin, *Griffonia (Bandeiraea) simplicifolia* Lectin I (Fluorescein Lectin Kit catalog no. FLK-4100) were purchased from Vector Laboratories, and Blue pseudo color (Draq5) was purchased from Cell Signaling Technology, Inc. (Beverly, MA, USA).

### 2.3. Lectin Histochemistry of the Heart

Histological slides containing up to 12 different heart samples each were deparaffinized using xylene and alcohol, followed by rinsing with distilled water. After two washes in distilled water, the slides were incubated for 60 min with specific lectins [19]. Following incubation, the samples underwent two further rinses in distilled water and were then stained with Draq5 for 5 min to visualize nuclei. All staining procedures were performed according to the manufacturers’ protocols.

This protocol was applied for the following lectins: ConA, WGA, RCA, BS, and Tomato [19]. To streamline the process across all sample groups, double staining was employed using ConA with Tomato and WGA with BS, ensuring that there was no signal overlap between the stains.

As a negative control, tissue sections were processed following the same protocol but incubated solely with the mounting medium, omitting lectins and all fluorescent dyes. This approach enabled assessment of the level and pattern of inherent tissue autofluorescence. To specifically address lipofuscin-related autofluorescence—especially relevant in aged samples, its presence was confirmed based on its characteristic broad-spectrum emission and distinctive perinuclear localization.

### 2.4. Confocal Scanning Laser Microscopy (CSLM) Analysis

Confocal fluorescence images were captured using a Zeiss LSM 710 confocal laser scanning module (Carl Zeiss Limited, Cambourne, United Kingdom) mounted on an Axio Observer Z1 Inverted Microscope (Carl Zeiss Limited, Cambourne, United Kingdom). To visualize cellular structures, excitation laser lines at 543 nm and 633 nm were used to detect Draq5 (emission bandpass [BP] 661–759 nm), Rhodamine (BP 548–629 nm), and Fluorescein (BP 514–521 nm), respectively. All images were acquired using a Zeiss Plan Apochromat 63× oil immersion objective (NA 1.4, DIC M27) (Carl Zeiss Limited, Cambourne, United Kingdom). Image acquisition, merging, processing, and analysis were conducted using the standard ZEN software version 2.3 provided by Zeiss. Fluorescence intensities were reported in arbitrary units (AU).

### 2.5. Lectins Signal Quantification by CLSM

The expression of Rhodamine-labelled ConA, WGA, and RCA, as well as Fluorescein-labelled Tomato and BS in heart tissue, was measured using an approach previously described by the authors’ group [57,58]. Quantitative analysis of the confocal images was performed automatically using the point-by-point fluorescence quantification functions available in the ZEN software.

To ensure consistent measurement conditions and high reproducibility of the data, confocal laser scanning microscopy (CLSM) image acquisition parameters were standardized and maintained throughout the experiment. The acquisition settings were as follows: acquisition time of 30 s, laser output power at 20%, pinhole diameter of 56 µm, master gain settings of 546 (Rhodamine), 771 (FITC), and 857 (DRAQ), digital gain of 15, numerical aperture (NA) of 1.4, scan zoom of 1.0, pixel dwell time of 10.035 µs, unidirectional scan mode, and 8-bit image depth [59,60].

Each image captured measured 512 × 512 pixels (135 × 135 µm), corresponding to an area of 18.225 µm^2^ per microscopic field. For quantification, mean fluorescence intensity was measured across five randomly selected microscopic fields, representing a total analysed area of 91.125 µm^2^.

### 2.6. Phyton Script

Using Python version 3.11.13, a script was developed to automate the process of extracting data from .lsm files. The script reads multi-channel confocal microscopy images, processes each channel individually, and compiles the statistical metrics—minimum, maximum, median, and standard deviation—into a single Excel file. The tiff file library was chosen due to its robust support for .lsm files and multi-dimensional TIFF formats, allowing the image data to be loaded as NumPy arrays for efficient analysis. Users can select a root folder containing .lsm files, after which the script automatically scans all subfolders, extracts image data from each file, computes basic statistics per channel, and exports the results to an Excel spreadsheet. The script can be found in Supplementary Materials.

### 2.7. Statistics

For each of the 64 cases analysed, myocardial tissue was systematically sampled from three predefined anatomical regions (LV, RV, At). To minimize variability, regions were identified using standardized anatomical landmarks, and equivalent locations were consistently selected across all cases. Within each region, four consecutive sections were prepared—one section served as a negative control, while the remaining three were stained with ConA & Tomato, WGA & BS, or RCA, respectively. This sampling design yielded a total of 768 sections (192 for each staining condition, including controls).

From each of these 768 sections, five non-overlapping high-power fields (HPFs) were randomly selected using a predefined grid-based randomization strategy. This ensured unbiased sampling while maintaining representative coverage of each anatomical region. In total, 3840 confocal microscopy micrographs were generated. For each HPF, descriptive statistics—including minimum, maximum, median, and standard deviation—were computed. To correct for potential artifacts and inter-regional variability, the median value of each stained section was normalized against the corresponding regional median obtained from its negative control. This normalization step ensured that only lectin-specific binding signals were carried forward into subsequent analyses.

Cases stratified along two dimensions: anatomical region (At, LV, RV) and heart weight, with the latter dichotomized into non-cardiomyopathy (heart weight < 18 g) and the HCM (heart weight ≥ 18 g).

Data distribution was assessed using the Shapiro–Wilk test. Because several variables were non-normally distributed and subgroup sizes were small, all subsequent analyses used non-parametric tests. Pairwise regional comparisons (median values per case) were conducted with the Mann-Whitney U test. Within the pathological (HCM) group, animals were classified into four age categories (kitten, young, mature, senior), and group differences were evaluated using the Kruskal-Wallis test with Dunn’s post hoc comparisons. To limit false positives across multiple regional and lectin-binding comparisons, Bonferroni-adjusted *p*-values were applied in post hoc testing.

All statistical analyses were performed in R (version 4.2.2), with statistical significance defined as *p* < 0.05. For all statistically significant results, effect sizes were additionally calculated to estimate the magnitude of observed differences. Eta squared (η^2^) was reported for Kruskal-Wallis tests, while the rank biserial correlation coefficient (r) was calculated for Mann-Whitney U tests. Effect sizes were interpreted according to Cohen’s guidelines (Table 2).

## 3. Results

### 3.1. Gross and Histological Findings

For the 2-day-old subject, the necropsy examination revealed that the myocardial walls were uniformly thin, consistent with the expected physiological anatomy of a neonate. The ventricular chambers were open and relaxed, suggesting a diastolic state. No macroscopic abnormalities such as hypertrophy, septal defects, or valve malformations were observed.

**Figure 1:**
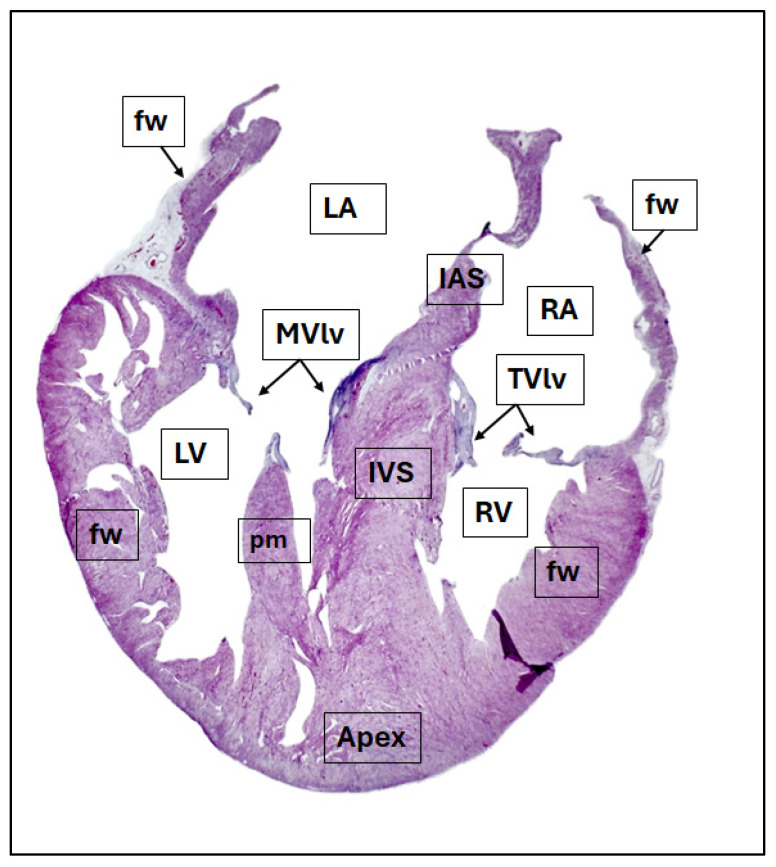
Histological section of the heart of the newborn subject. The heart was sectioned using the four-chamber dissection technique, through which all the components of the heart can be observed. Hematoxylin & Eosin stain. LA—left atrium, MVlv—mitral valve, LV—left ventricle, pm—papillary muscle, IAS—interatrial septum, IVS—interventricular septum, RA—right atrium, TVlv—tricuspid valve, RV—right ventricle, fw—free wall.

**Figure 2:**
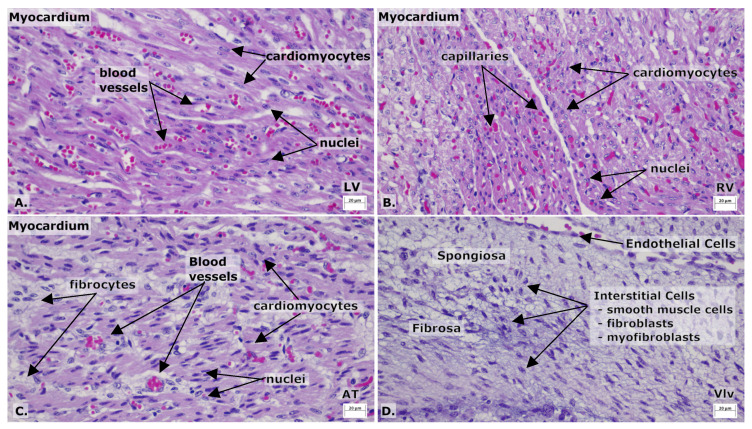
Normal structural organization of the myocardium and cardiac valve from a newborn kitten. (**A**) The left ventricle free wall displayed densely packed cardiomyocytes with centrally located, oval nuclei, interspersed with small-caliber blood vessels within a scant connective tissue stroma. (**B**) The right ventricle free wall showed cardiomyocytes arranged in branching fibers and containing centrally placed nuclei; capillaries were observed within the endomysial connective tissue. (**C**) The left atrium demonstrated a thinner myocardial layer compared to the ventricles, composed of elongated cardiomyocytes, fibrocytes, and small blood vessels within the interstitial tissue. (**D**) The tricuspid valve exhibited the characteristic layered organization: the fibrosa, rich in collagen fibers; the spongiosa, containing loose connective tissue; and the ventricularis, lined by endothelial cells. Interstitial cells, including smooth muscle cells, fibroblasts, and myofibroblasts, were identified within the valve matrix. H&E stain; ob ×40; scale bare = 20 μm.

Histologically, the myocardium was composed of densely arranged, immature cardiomyocytes with relatively high nuclear-to-cytoplasmic ratios, indicative of neonatal cardiac tissue. Cross-striations were poorly defined, and intercalated discs were not prominent (findings typical of early postnatal myocardial development). There was no evidence of myofiber disarray, interstitial fibrosis, necrosis, or inflammatory infiltrates. The endocardium and epicardium were intact. The mitral and tricuspid valve leaflets appeared thin and translucent, with no histological changes suggestive of myxomatous degeneration or endocarditis. These findings were consistent with a normal neonatal cardiac phenotype and were used as a comparative reference for the identification of pathological changes in diseased hearts included in the study (Figure 1 and Figure 2).

### 3.2. Confocal Scanning Laser Microscopy (CSLM)

#### 3.2.1. Left Ventricle

In healthy LVs, age-related variation was most apparent for ConA, WGA, and Tomato lectins. For ConA, newborns displayed a predominantly membrane-associated signal, whereas adult and senior cats exhibited a more cytoplasmic distribution, indicating an age-related redistribution of high-mannose N-glycans. Senior cats demonstrated increased ConA binding relative to younger age groups. Tomato lectin binding followed a similar age-associated rise, with seniors showing markedly elevated levels compared to all younger groups. In contrast, WGA exhibited a decline in seniors relative to kittens, indicating an age-related reduction of GlcNAc- or sialic acid–containing residues in normal LV tissue.

In left ventricular tissue of senior cats with HCM, ConA binding was markedly reduced, consistent with lower detection of high-mannose N-glycans. The Tomato lectin binding was also diminished, indicating reduced detection of poly-GlcNAc sequences often present on extracellular matrix components and adhesion molecules. These decreases were most evident in older animals and in samples representing more advanced disease stages.

The RCA binding remained relatively consistent across most groups but showed a significant reduction of terminal β-D-galactose between young and mature HCM cats.

**Figure 3:**
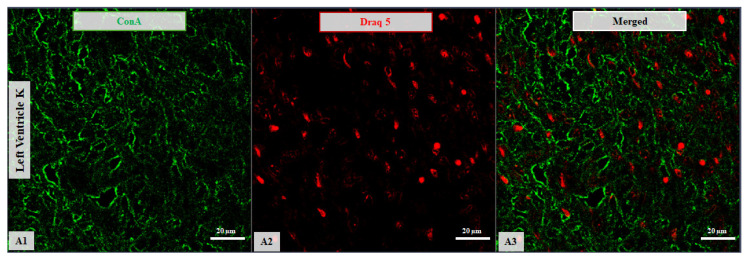
Confocal microscopy micrographs of left ventricular tissue from a 2-day-old newborn. The ConA expression (green channel, **A1**) shows affinity for the cellular membrane of the LV. Nuclei are stained with DRAQ5 (red channel, **A2**). Merged images are shown for all samples (**A3**). Images were acquired using a 63×/1.4 Oil Plan Apochromat objective.

**Figure 4:**
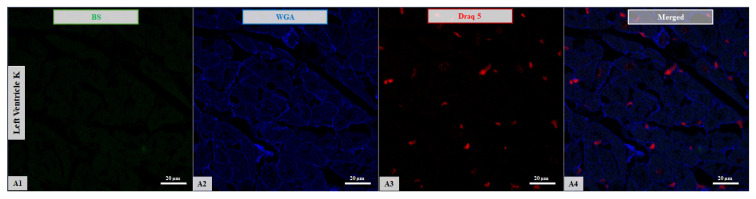

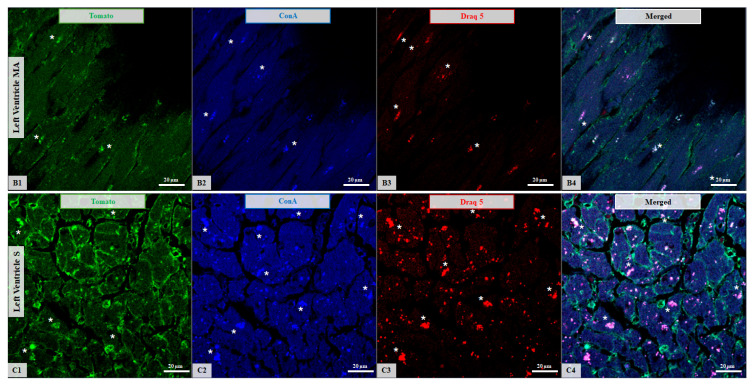
Confocal microscopy micrographs of left ventricular tissue from cats in control group. (**A**) Comparison of BS expression (green channel, **A1**) with WGA expression (blue channel, **A2**) in a 6-month-old kitten. The BS expression is nearly absent, while WGA shows preferential binding to the cellular membrane. (**B**) Comparison of Tomato expression (green channel, **B1**) with ConA expression (blue channel, **B2**) in a 7-year-old mature adult. The Tomato shows strong membrane localization, whereas ConA displays cytoplasmic affinity. Sporadic lipofuscin (asterisk *) accumulation is observed in the cytoplasm and is visible across all channels (**B1**–**B4**). (**C**) Comparison of Tomato expression (green channel, **C1**) with ConA expression (blue channel, **C2**) in a 14-year-old senior cat. The Tomato shows strong affinity for both the cellular membrane and vascular wall, while ConA displays more pronounced cytoplasmic localization (**B2**). Marked lipofuscin (asterisk *) accumulation is present in the cytoplasm, visible across all channels (**C1**–**C4**). Nuclei are stained with DRAQ5 (red channel; **A3**,**B3**,**C3**). Merged images are shown for all samples (**A4**,**B4**,**C4**). Images were acquired using a 63×/1.4 Oil Plan Apochromat objective.

Overall, the findings indicate that lectin-binding profiles in the LV differ with both age and HCM status. Representative confocal images in Figure 3 and Figure 4 illustrate these patterns along with increased lipofuscin accumulation in samples from older subjects.

#### 3.2.2. Right Ventricle

In healthy RVs, all lectins except RCA showed age-related differences, indicating developmental and aging-related variation in profiles of high-mannose, sialylated/GlcNAc, poly-GlcNAc, and α-galactosyl/N-acetylgalactosamine glycans. ConA binding was highest in kittens and progressively decreased with age. Tomato lectin binding also peaked in kittens and declined across subsequent age groups. WGA binding was elevated in young healthy animals relative to mature adults, and BS lectin showed a similar pattern, with higher binding in young cats than in mature individuals.

In HCM-affected right ventricular tissue, lectin binding showed distinct age-dependence. ConA binding was significantly reduced in young animals but increased in seniors relative to controls. Tomato lectin showed the same pattern, with lower binding in young and higher binding in senior HCM hearts. WGA binding was notably elevated in young HCM animals, while BS lectin binding increased in both young and senior groups, indicating age- and disease-related shifts in glycan profiles.

**Figure 5:**
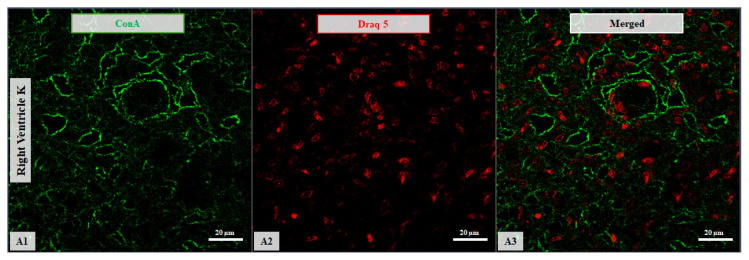
Confocal microscopy micrographs of right ventricular tissue from a 2-day-old newborn. The ConA expression (green channel; **A1**) shows affinity for the cellular membrane of the RV. Nuclei are stained with DRAQ5 (red channel; **A2**). Merged images are shown for all samples (**A3**). Images were acquired using a 63×/1.4 Oil Plan Apochromat objective.

**Figure 6:**
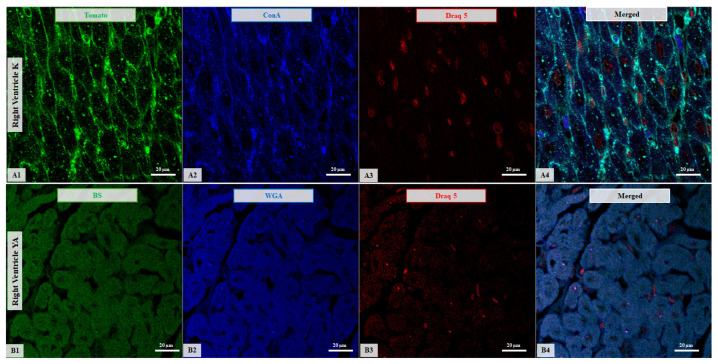
Confocal microscopy micrographs of the right ventricular tissue from cats. (**A**) Comparison of Tomato expression (green channel, **A1**) with ConA expression (blue channel, **A2**) in a 6-month-old kitten. Both lectins exhibit strong membrane localization. (**B**) Comparison of BS expression (green channel, **B1**) with WGA expression (blue channel, **B2**) in a 3-year-old young adult with HCM, where both lectins show moderate cytoplasmic affinity. Nuclei are stained with DRAQ5 (red channel; **A3**,**B3**). Merged images are shown for all samples (**A4**,**B4**). Images were acquired using a 63×/1.4 Oil Plan Apochromat objective.

Together, these results show that lectin-binding profiles in the RV differ with both age and HCM status. Representative examples are shown in Figure 5 and Figure 6, which depict confocal micrographs of right ventricular sections across age and disease groups, illustrating variations in binding intensity, subcellular distribution (membrane vs. cytoplasmic), and occasional regional differences.

#### 3.2.3. Atrium

In healthy atria, age-related variation was most evident for Tomato, WGA, and BS lectins. For Tomato and BS lectin, the strongest labelling exhibited in kittens and young animals and declined progressively in mature and senior groups, reflecting developmental and aging-associated remodelling of poly-GlcNAc, sialylated/GlcNAc, and α-galactosyl/N-acetylgalactosamine-containing glycans. While WGA displayed a distinctive pattern marked by high binding in kittens, a transient decrease in mature animals, and renewed enhancement in senior atria.

In atrial tissue from young and senior HCM cats, ConA binding was significantly reduced, indicating lower detection of high-mannose N-glycans, while RCA binding was also decreased in young HCM samples, reflecting reduced detection of terminal β-D-galactose residues.

**Figure 7:**
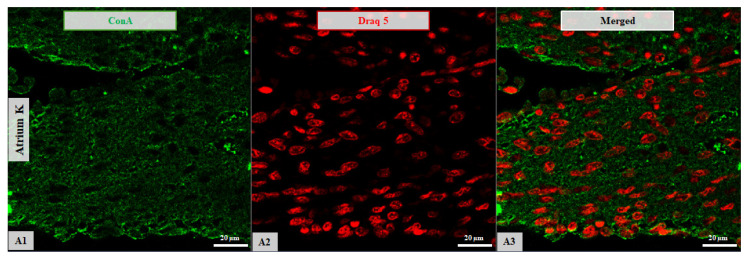
Confocal microscopy micrographs of atrial tissue from a 2-day-old newborn. The ConA expression (green channel; **A1**) shows affinity for both the cellular membrane and cytoplasm of the AT. Nuclei are stained with DRAQ5 (red channel; **A2**). Merged images are shown for all samples (**A3**). Images were acquired using a 63×/1.4 Oil Plan Apochromat objective.

**Figure 8:**
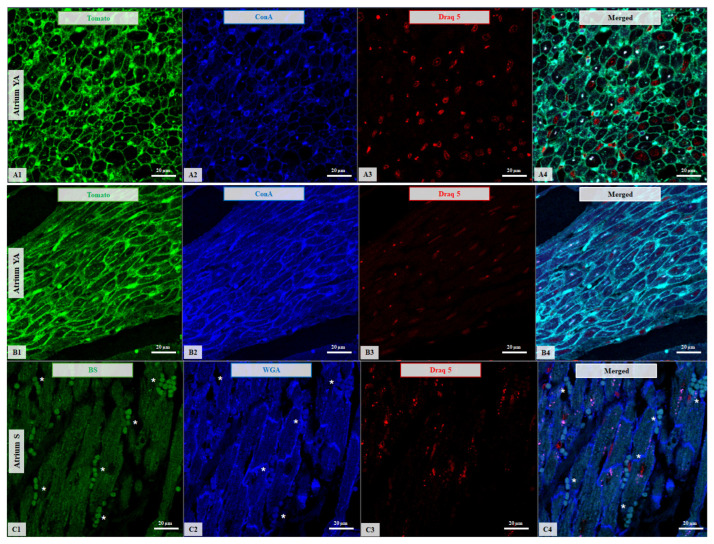
Confocal microscopy micrographs of atrial tissue from cats. (**A**) Comparison of Tomato (green channel, **A1**) and ConA (blue channel, **A2**) expression in a 4-year-old young adult with HCM. Both lectins bind to the cellular membrane, with Tomato exhibiting more intense binding. (**B**) Comparison of Tomato (green channel, **B1**) and ConA (blue channel, **B2**) expression in a 5-year-old young adult with HCM. Both lectins show affinity for both the cellular membrane and cytoplasm, with stronger membrane localization. (**C**) Comparison of BS expression (green channel, **C1**) and WGA expression (blue channel, **C2**) in a 12.5-year-old senior cat. The BS shows low membrane affinity and higher binding to red blood cells (asterisk *) (**C1**,**C4**), while WGA demonstrates strong membrane affinity and moderate red blood cells (asterisk *) and cytoplasmic localization (**C2**,**C4**). Sporadic lipofuscin accumulation is present in the cytoplasm, visible across WGA and Draq5 channels as high intensity points (**C2**,**C3**) and can be observed as magenta points (**C4**). Nuclei are stained with DRAQ5 (red channel; **A3**,**B3**,**C3**). Merged images are shown for all samples (**A4**,**B4**,**C4**). Images were acquired using a 63×/1.4 Oil Plan Apochromat objective.

Overall, atrial lectin-binding patterns varied across both age and HCM status. Representative examples are shown in Figure 7 and Figure 8, illustrating variations in binding intensity, subcellular distribution (membrane versus cytoplasmic), and occasional red blood cell staining across age and disease groups.

### 3.3. Statistical Analysis

To evaluate the lectin affinity across different age groups within the heart (Senior, Mature, Young, Kitten), the Mann-Whitney U tests were performed for each lectin (ConA, RCA, Tomato, WGA, BS). Also, the Kruskal-Wallis tests followed by Dunn’s multiple comparisons with Bonferroni correction were conducted to assess differences across age groups in the HCM group and for the control group. For comparison of the statistical significance evaluation the effect size was also assessed. These tests were conducted for all the heart components—LV, RV, AT.

#### 3.3.1. Left Ventricle

A. General Normal Group Analyses (Kruskal-Wallis tests)

**Figure 9:**
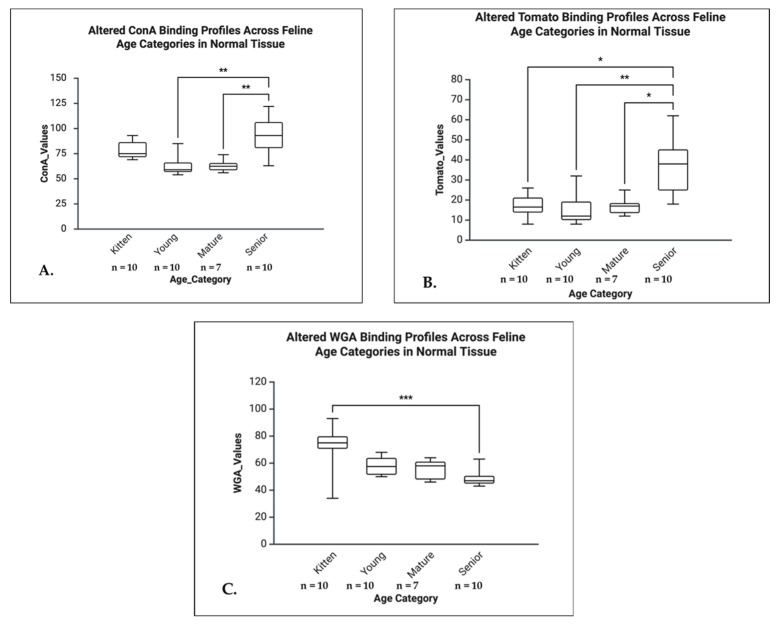
Lectin binding profiles across feline age categories in normal left ventricular tissue. (**A**) The ConA binding is higher in Kitten and Senior cats, with Seniors showing the greatest range and highest overall values. Young and Mature groups exhibit lower, more clustered levels. Seniors display significantly increased ConA binding relative to Young and Mature cats (*p* < 0.01), suggesting age-associated shifts in myocardial glycoprotein composition. (**B**) The Tomato lectin binding shows a sharp increase in seniors, potentially reflecting age-associated changes in glycoprotein composition. (**C**) The WGA binding in normal tissue increases steadily with age, contrasting with the decline seen in HCM, possibly indicating distinct aging mechanisms or compensatory glycosylation in healthy myocardium. * *p* < 0.05, ** *p* < 0.01, *** *p* < 0.001.

Significant age-related differences in lectin binding were observed for ConA (H = 19.807, *p* < 0.001, η^2^ = 0.53), Tomato (H = 14.853, *p* = 0.002, η^2^ = 0.37), and WGA (H = 15.622, *p* = 0.001, η^2^ = 0.41), all indicating large effect sizes. For ConA, Dunn’s post hoc analysis revealed significantly higher binding in seniors compared to mature (*p* = 0.004, Δ = +17.40) and young animals (*p* = 0.001, Δ = +18.39). The Tomato lectin also showed increased binding in seniors compared to mature (*p* = 0.043, Δ = +13.76), young (*p* = 0.002, Δ = +17.54), and kittens (*p* = 0.032, Δ = +13.83). In contrast, WGA binding was significantly reduced in seniors compared to kittens (*p* < 0.001, Δ = −18.39). No significant age-related differences were observed for RCA and BS (Figure 9).

B. General HCM Group Analyses (Kruskal-Wallis tests)

**Figure 10:**
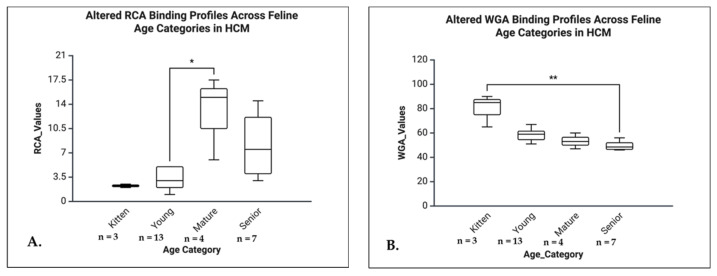
Lectin binding profiles across feline age categories in the LV in HCM cardiac tissue. (**A**) RCA binding is lowest in kittens and young cats, peaks significantly in the mature group (*p* < 0.05), and decreases in seniors, indicating age-related alterations in galactose-containing glycoconjugates. (**B**) The WGA binding in HCM-affected LVs shows a pronounced age-dependent decline, indicating a reduction in GlcNAc and/or sialic acid residues in older animals. * *p* < 0.05, ** *p* < 0.01.

Significant age-related differences were identified for RCA (H = 11.610, *p* = 0.009, η^2^ = 0.45) and WGA (H = 13.014, *p* = 0.005, η^2^ = 0.56), both showing large effect sizes. Post hoc Dunn’s test revealed a significant decrease in RCA binding between mature and young animals (*p* = 0.037, Δ = −11.91). For WGA, binding was significantly increased in kittens compared to seniors (*p* = 0.004, Δ = +16.10). No significant age-related changes were observed for ConA, Tomato, or BS (Figure 10).

C. Age Group Analyses (Mann-Whitney U tests)

**Figure 11:**
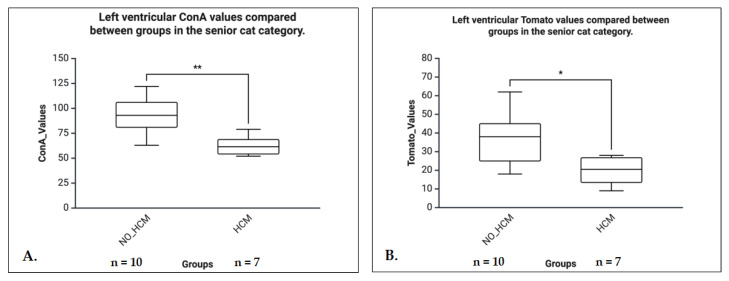
Left ventricular ConA and Tomato lectin binding in senior cats, comparing NO-HCM (control) and HCM groups. (**A**) The ConA binding values were significantly higher in the non-HCM group compared to the HCM group, indicating reduced mannose/glucose residue availability in the LV of cats with HCM. (**B**) The Tomato lectin binding was also higher in the non-HCM group, suggesting decreased N-acetylgalactosamine or galactose expression in the HCM-affected myocardium.* *p* < 0.05, ** *p* < 0.01.

In senior cats, lectin binding was significantly reduced in HCM hearts for ConA (U = 4.0, *p* = 0.005, r = 0.70) and Tomato (U = 9.0, *p* = 0.036, r = 0.55), both indicating large effect sizes. No other significant differences were observed, although ConA (*p* = 0.064) and Tomato (*p* = 0.052) in the mature group, and RCA in kittens (*p* = 0.056), showed trends toward significance (Figure 11).

#### 3.3.2. Right Ventricle

A. General Normal Group Analyses (Kruskal-Wallis tests)

**Figure 12:**
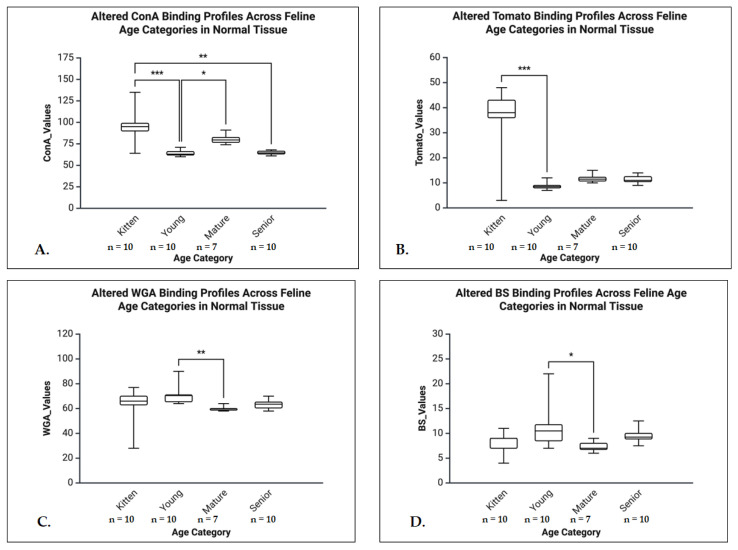
Age-related alterations in lectin binding profiles in the RV of normal feline hearts. (**A**) The ConA binding was significantly higher in kittens compared to young (*p* < 0.001), and senior cats (*p* < 0.05), and higher binding intensity in mature compared to young indicating a progressive reduction of α-mannosyl and glucose-containing structures with age. (**B**) The Tomato lectin binding was highest in kittens and declined significantly across all older age groups (*p* < 0.001), suggesting age-related reductions in poly-N-acetyl-lactosamine or galactosyl residue availability. (**C**) The WGA binding significantly decreased in mature cats compared to young cats (*p* < 0.01), indicating reduced GlcNAc and/or sialic acid residues with age. (**D**) BS-I binding was also significantly reduced in mature cats compared to young cats (*p* < 0.05), reflecting age-associated alterations in α-galactosyl expression.* *p* < 0.05, ** *p* < 0.01, *** *p* < 0.001.

Kruskal-Wallis tests revealed significant age-related glycosylation differences in the normal group, with post hoc Dunn’s tests (Bonferroni-corrected) identifying key contrasts. The ConA showed a strong age effect (H = 22.95, *p* < 0.001, η^2^ = 0.67), with significant differences between kittens and young (*p* < 0.001), mature and young (*p* < 0.026), and between kitten and senior (*p* = 0.01). The Tomato lectin also demonstrated a large age effect (H = 17.892, *p* < 0.001, η^2^ = 0.50), with increased binding in kittens vs. young (*p* < 0.001). The WGA showed a moderate to large effect (H = 12.790, *p* = 0.005, η^2^ = 0.38), with an increased binding in young vs. mature (*p* = 0.005). The BS lectin showed a low age effect (H = 10.030, *p* = 0.018, η^2^ = 0.26), with increased binding in young vs. mature (*p* = 0.034). The RCA showed no age-related variability (Figure 12).

B. General HCM Group Analyses (Kruskal-Wallis tests)

**Figure 13:**
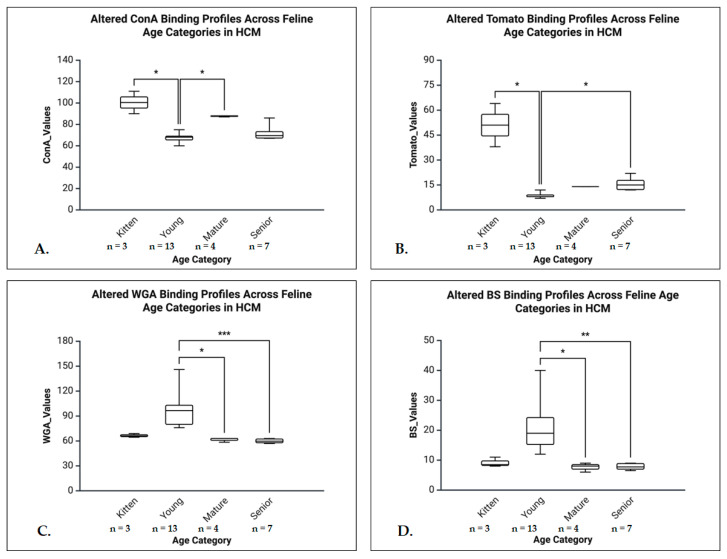
Age-related alterations in lectin binding in the RV of cats with HCM. (**A**) Box plot showing ConA binding intensity in the right ventricular myocardium across feline age groups: kitten, young, mature, and senior. The ConA binding was significantly higher in kittens compared to young cats and in mature cats compared to young cats (*p* < 0.05), suggesting an age-related modulation of α-D-mannosyl and α-D-glucosyl residues, with higher early-life and mature levels and a relative decline during the young developmental stage. (**B**) The Tomato lectin binding was significantly higher in kittens than in young and in senior than in young cats (*p* < 0.05), suggesting an age-dependent modulation of poly-N-acetyl-lactosamine or galactosyl residues, characterized by high early-life expression, a decline during maturity, and a secondary increase in advanced age associated with myocardial remodelling in HCM. (**C**) The WGA binding peaked in the young group and was significantly higher than in the mature (*p* < 0.05) and senior (*p* < 0.001) groups, reflecting potential age-dependent modulation of GlcNAc and sialic acid residues during HCM progression. (**D**) The BS binding was significantly elevated in young cats compared to mature (*p* < 0.05) and senior cats (*p* < 0.01), indicating dynamic regulation of β-D-galactosyl residues during disease development. * *p* < 0.05, ** *p* < 0.01, *** *p* < 0.001.

The tests revealed significant age-related glycosylation changes in HCM hearts, identifying key differences. The ConA showed a large effect (H = 12.360, *p* = 0.006, η^2^ = 0.52), with significant differences between kittens and young (*p* = 0.036) and mature vs. young animals (*p* = 0.048). The Tomato lectin demonstrated a large size age effect (H = 16.772, *p* < 0.001, η^2^ = 0.77), with higher binding in kittens vs. young (*p* = 0.011) and senior vs. young (*p* = 0.017). The WGA also showed a large effect (H = 19.090, *p* < 0.001, η^2^ = 0.80), with an increased binding in young vs. mature (*p* = 0.036) and young vs. senior animals (*p* < 0.001). The BS lectin revealed a significant age effect (H = 17.631, *p* < 0.001, η^2^ = 0.73), with young animals showing higher binding compared to both mature (*p* = 0.026) and senior individuals (*p* = 0.003). The RCA was excluded due to lack of variability across age groups (Figure 13).

C. Age Group Analyses (Mann-Whitney U tests)

**Figure 14:**
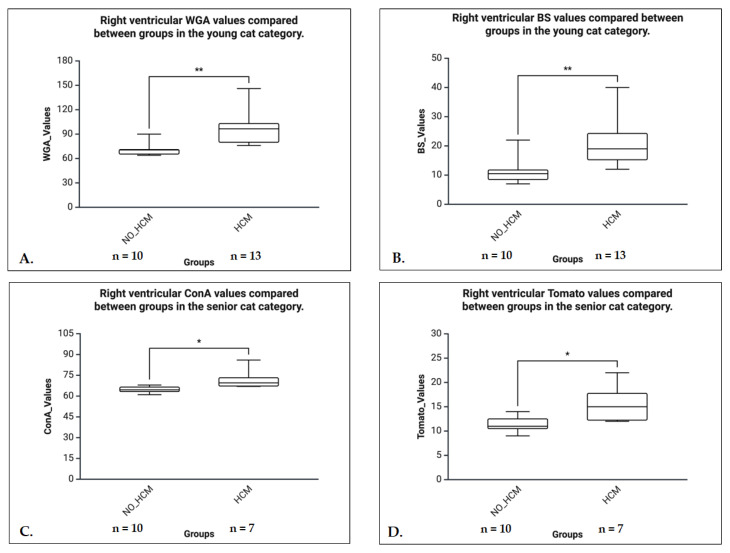
Right ventricular lectin binding in young (**A**,**B**) and senior cats (**C**,**D**), comparing non-HCM and HCM groups. (**A**) The WGA binding in the RV of young cats was significantly higher in the HCM group compared to non-HCM controls (*p* < 0.01), indicating increased glycoprotein expression or accessibility in the diseased myocardium. (**B**) The BS lectin binding in young cats was significantly elevated in the HCM group compared to non-HCM cats (*p* < 0.01), suggesting alterations in terminal α-galactosyl residues associated with HCM. (**C**) The ConA binding in the RV of senior cats was significantly greater in the HCM group than in non-HCM controls (*p* < 0.05), indicating changes in mannose/glucose residue presentation in the diseased myocardium. (**D**) The Tomato lectin binding in senior cats was significantly increased in the HCM group compared to non-HCM controls (*p* < 0.05), reflecting altered glycosylation patterns in the right ventricular myocardium. * *p* < 0.05, ** *p* < 0.01.

In senior animals, HCM hearts showed significantly higher expression for ConA (U = 5.000, *p* = 0.016, r = 0.66), Tomato (U = 7.000, *p* = 0.031, r = 0.59), and BS (U = 7.500, *p* = 0.036, r = 0.57), all with large effect sizes. The WGA was not significant (U = 9.000, *p* = 0.100), and RCA was excluded due to identical values. In the young group, significant higher expressions in HCM hearts were noticed for WGA (U = 4.000, *p* = 0.003, r = 0.71) and BS (U = 7.500, *p* = 0.009, r = 0.63), both with large effect sizes. No significant differences were observed in mature or kitten groups for any lectin. Due to lack of variability, RCA could not be analyzed in any group (Figure 14).

#### 3.3.3. Atrium

A. General Normal Group Analyses (Kruskal-Wallis tests)

**Figure 15:**
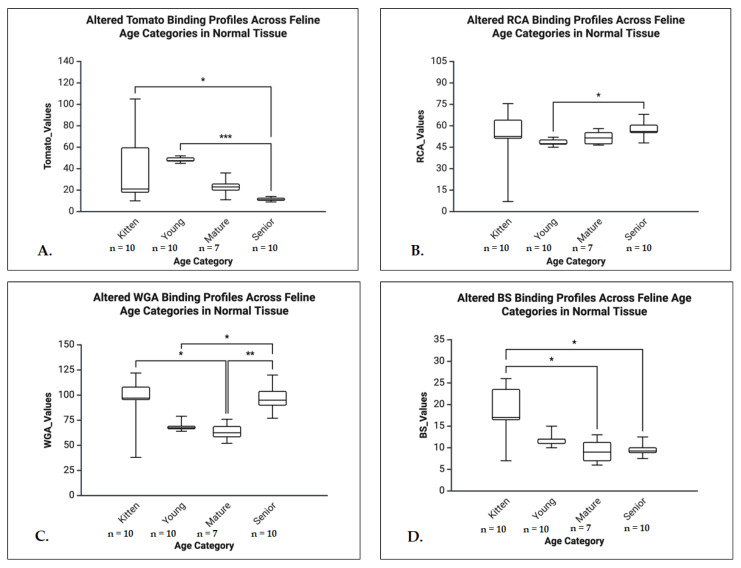
Age-dependent variation in lectin binding profiles in feline normal atrial tissue. (**A**) The Tomato lectin binding was highest in kittens and significantly declined with age, with a marked decrease between Young and Senior (*** *p* < 0.001) and between Kitten and Senior cats (* *p* < 0.05). (**B**) The RCA binding differed significantly between Senior and Young groups (* *p* < 0.05). (**C**) The WGA binding exhibited a biphasic pattern with a decline from Kitten to Mature and a rebound in Seniors; significant differences were observed across multiple comparisons (* *p* < 0.05, ** *p* < 0.01). (**D**) The BS lectin binding decreased progressively with age, with kittens showing significantly higher values than the other age categories (* *p* < 0.05).

Besides ConA, which showed no significant differences, all the other lectins revealed significant age-related changes in lectin binding across several markers, followed by post hoc comparisons to identify specific group differences. The RCA demonstrated a significant overall age effect (H = 11.610, *p* = 0.009, η^2^ = 0.31, indicating a large effect size). Post hoc analysis revealed that senior animals showed significantly increased RCA binding compared to young animals (*p* = 0.048, Δ = 12.06). The Tomato lectin binding varied significantly with age (H = 14.853, *p* = 0.002, η^2^ = 0.40, large effect size). Specifically, senior animals had significantly reduced binding levels compared to both kittens (*p* = 0.035, Δ = 13.77) and young animals (*p* < 0.001, Δ = 19.56). The WGA showed the strongest age-related effect (H = 15.622, *p* = 0.001, η^2^ = 0.47). Kittens had significantly higher WGA binding compared to mature group (*p* = 0.013, Δ = 14.84). While in senior animals were observed significantly higher bindings levels compared to mature and young, suggesting a progressive increase with age. The BS lectin also exhibited significant age-related differences (H = 10.030, *p* = 0.018, η^2^ = 0.25, small to moderate effect). Binding levels were significantly higher in kittens compared to both seniors (*p* = 0.048, Δ = 12.06) and mature (*p* = 0.0037, Δ = 15.56) (Figure 15).

B. General HCM Group Analyses (Kruskal-Wallis tests)

**Figure 16:**
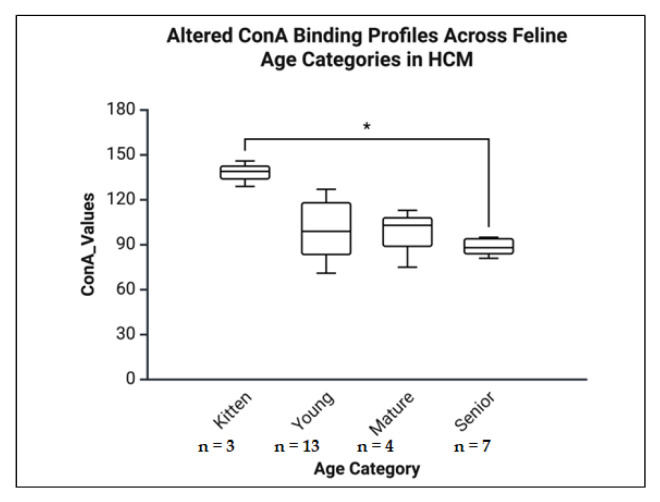
Atrial ConA binding profiles across feline age categories in the HCM group. Boxplot representation of ConA binding values in atrial tissue from cats diagnosed with HCM, stratified by age category: kitten, young, mature, and senior. A significant decrease in ConA binding was observed from kittens to senior cats (*p* < 0.05), indicating age-related alterations in glycosylation patterns in the AT of HCM-affected animals. * *p* < 0.05.

The ConA exhibited a significant age-related effect (H = 8.582, *p* = 0.035, η^2^ = 0.29, indicating a large effect size), with Dunn’s post hoc test revealing a significant increase in binding in kittens compared to seniors (*p* = 0.025, Δ = +14.20). Although RCA and Tomato lectins showed overall statistical significance (*p* = 0.030 and *p* = 0.015, respectively), no significant differences were detected in pairwise comparisons. In contrast, WGA and BS did not demonstrate any significant age-related changes (Figure 16).

C. Age Group Analyses (Mann-Whitney U tests)

**Figure 17:**
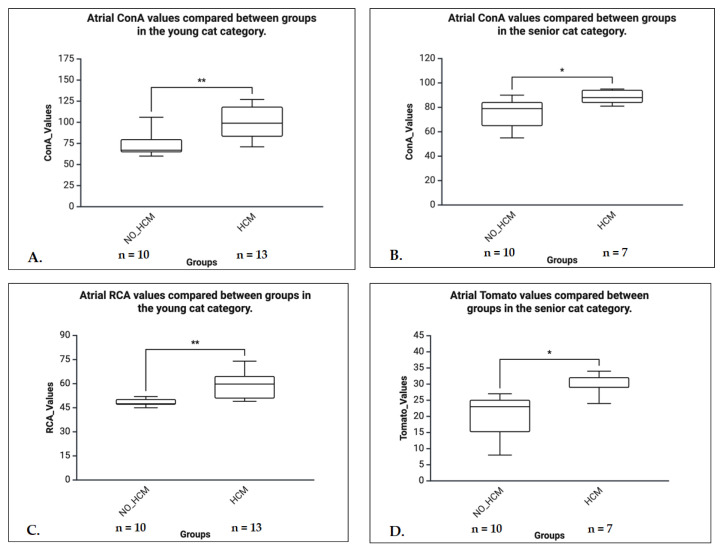
Boxplots illustrating differences in atrial lectin binding values between control (NO-HCM) and HCM groups in young and senior cats. (**A**) The ConA binding values in young cats. HCM cats showed significantly higher ConA values compared to NO-HCM (*p* < 0.01). (**B**) The ConA values in senior cats. A moderate but significant elevation in ConA binding was seen in HCM compared to NO-HCM (*p* < 0.05). (**C**) The RCA binding values in young cats. A significant increase was observed in the HCM group compared to NO-HCM (*p* < 0.01). (**D**) The Tomato lectin binding values in senior cats. The HCM cats exhibited significantly higher values than NO-HCM (*p* < 0.05).* *p* < 0.05, ** *p* < 0.01.

In the senior group, significant differences in lectin binding between HCM and control animals were observed for ConA (U = 6.500, *p* = 0.038, r = 0.57) and Tomato lectin (U = 3.500, *p* = 0.019, r = 0.68), both indicating a large effect sizes. In the young group, lectin binding was significantly reduced in the control group for both ConA (U = 20.000, *p* = 0.005, r = 0.59) and RCA (U = 10.500, *p* = 0.001, r = 0.70), also with large effect sizes. No significant differences were observed in the mature or kitten groups (Figure 17).

#### 3.3.4. Integrated Analysis of Lectin Binding Patterns in Feline Cardiac Tissue

**Figure 18:**
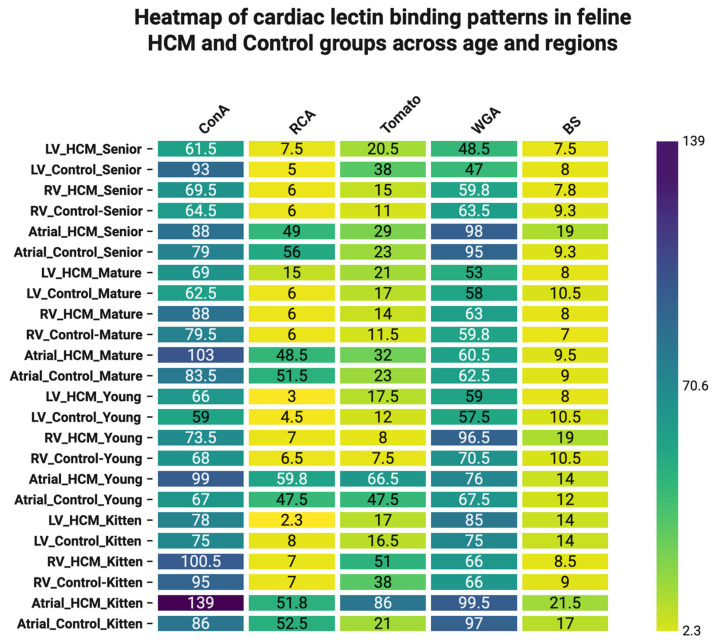
Global Heatmap of Lectin Binding Patterns Across Feline Cardiac Tissues by Age and Disease Status. This heatmap illustrates the average binding intensities of five lectins (ConA, RCA, Tomato, WGA, and BS) across feline cardiac tissues (LV, RV, and AT) grouped by age category (kitten, young, mature, senior) and disease status (HCM vs. control). Each row represents a tissue–age–condition combination, and each column corresponds to a lectin. Warmer colors (yellow) indicate reduced lectin affinity, while cooler colors (blue–purple) indicate higher binding intensities. The color scale ranges from low binding intensity (2.3) in bright yellow to high binding intensity (139) in deep purple, as shown in the scale bar. The heatmap’s hierarchical clustering reveals distinct glycosylation profiles linked to age-related changes and HCM pathology. The ConA, WGA, and Tomato exhibit the most pronounced variability across conditions.

In order to summarize the dataset and identify underlying patterns, a heatmap was generated to illustrate the average lectin binding intensity across various cardiac tissues, age groups, and disease conditions (Figure 18).

## 4. Discussion

This descriptive study examined lectin-binding profiles across different cardiac chambers of cats of varying ages, with and without HCM, providing preliminary insight into potential age- and disease-associated glycosylation shifts in the feline myocardium.

Lectin histochemistry remains a practical approach for cross-species comparison since lectins recognise conserved sugar motifs independent of species-specific antibody limitations. Lectin histochemistry is also generally more cost-effective, and the widespread availability of fluorophore-conjugated lectins reduces the number of experimental steps compared to traditional immunohistochemistry [40].

The ConA, which binds α-D-mannose and α-D-glucose residues, showed distinct binding patterns across cardiac chambers in HCM-affected and aged control cats. In the control ventricles, a progressive decline was observed across the age groups, with the exception of the senior LVs which exhibited an increased binding pattern. In the HCM group, both LVs and RVs showed a gradual decrease from kittens to seniors, with slight increase noted in the mature group. These patterns indicate that both aging and HCM are associated with altered ConA binding, but the direction of change varies by chamber. Comparable declines in mannose-rich glycans have been reported in murine and rat hearts [38,39,43], while zebrafish and giant danio hearts show regional variation, with stronger ConA staining in compact myocardium and coronary vessels than in trabecular zones [17]. Increased mannose-rich glycans have also been observed in vascular endothelium under inflammatory stress, highlighting tissue- and condition-specific variability [44]. In the current study, specific regional affinities resembled those seen in healthy feline kidneys [62]. While possible explanations such as altered glycoprotein synthesis, glycan processing, or extracellular matrix composition could account for these changes, the present study does not provide molecular evidence to confirm these mechanisms [39,43,46].

The WGA, which binds GlcNAc and sialic acid residues, displayed both chamber- and group-specific patterns. In the LVs, WGA binding progressively decreases with age in both control and HCM groups. In the RVs, binding peaked in young animals in both groups and declined with age. Across control and HCM groups, the ATs showed the highest binding in kittens and seniors, with significant lower values in young and mature subjects. These findings suggest that age and HCM influence WGA binding along distinct, chamber-specific trajectories. In the study dataset, young HCM cats showed higher WGA signal than age-matched controls, suggesting early upregulation of sialylated glycans under myocardial stress. Previous work has established WGA as a marker of sarcolemmal glycoproteins, cardiomyocyte boundaries, and cross-sectional area, sometimes substituting for anti-laminin immunohistochemistry [22,40,45]. Previous studies have reported age-related increases in GlcNAc- and sialic acid-binding lectins in murine models and in studies of cardiac development and stress in other species [38,39,43]. The WGA-binding GlcNAc residues, especially O-GlcNAc, are known to respond to cellular stress [46]. Whereas acute oxidative stress models report O-GlcNAc upregulation, the present results show reduced WGA binding in older HCM ventricles. This contrast may reflect differences in glycosylation patterns between chronic versus acute stress contexts, but mechanistic interpretation is speculative without confirmation. Unlike the relatively consistent WGA seen in non-cardiac tissues such as the kidney [62], cardiac WGA binding showed marked variability across chambers and disease states.

RCA, which binds β-D-galactose residues, showed stable and reduced binding across ventricles in all groups, but with high binding patterns were identified in ATs from both control and HCM groups. The lowest binding was observed in the LV and AT of young HCM cats, providing descriptive region-specific variations associated with early disease. Reduced RCA signal may reflect decreased exposure of Galβ1–4GlcNAc motifs due to increased sialylation, a mechanism previously reported in developmental masking of galactose residues in rodent hearts [38,47]. The tissue- and species-specific variability of RCA binding is highlighted by strong staining in zebrafish compact myocardium and coronary vessels [17], and by relatively stable RCA expression in healthy feline kidneys [62]. In murine hearts, galactose exposure increased only after sialidase treatment, suggesting condition-dependent masking [39]. Furthermore, loss of RCA-detectable glycans has been demonstrated in a model of impaired UDP-galactose biosynthesis, reversible with galactose supplementation [48]. Although the authors did not assess such metabolic mechanisms, the results provide descriptive evidence of chamber-specific variation in RCA binding in relation to HCM in young cats.

The Tomato lectin, a marker for poly-GlcNAc ([GlcNAc]1–3) structures, exhibited both age- and HCM-associated differences in the feline heart. In control RVs and ATs, binding was highest in the younger groups and declined progressively with age, whereas in control LVs the seniors group showed a significant increase. In HCM hearts, senior LVs exhibited reduced binding, while senior RVs and ATs showed increased binding compared with their respective controls, indicating disease-specific remodeling that differs by chamber. Confocal microscopy revealed a shift from predominantly membrane-associated labeling in young hearts to diffuse cytoplasmic staining in older and HCM hearts, suggesting altered glycan localization or tissue composition, although the underlying pathways remain unclear. Age-related reductions in chitin-binding lectins, including Tomato lectin, have been previously reported in specific regions of the aging murine heart [39], indicating partial conservation across species. In giant danio, Tomato lectin binds strongly to compact myocardium and valves but weakly to spongy myocardium and zebrafish ventricles [17], underscoring regional and species-specific variability. Beyond the heart, Tomato lectin is widely used as a vascular marker in rodents and humans, and other species [18,41,63]. In this study, the variability signal observed across different ages and in HCM hearts may reflect changes in vascular or myocardial glycosylation; despite this, potential contributions from altered glycan branching, glycosyltransferase activity, or extracellular matrix remodelling remain speculative without molecular validation.

The BS lectin, which binds α-galactosyl and GalNAc-containing glycans, showed both age- and HCM-associated differences in the feline heart. In healthy tissue, BS binding was highest in kittens and young animals in both the RV and AT and declined with age. In HCM-affected tissue, young RV samples showed significantly increased BS binding compared with age-matched controls. In the AT, senior HCM animals displayed a notable increase in BS binding relative to controls. Similar age-related declines in BS-IB4 binding have been reported in aging murine hearts, while strong BS reactivity is characteristic of metabolically active tissues such as placenta [39,42]. The region-specific staining patterns observed also align with species-dependent BS lectin reactivity described in fish cardiac tissues [17]. In other models, reduced BS binding has been linked to alterations in endothelial glycosylation, glycoprotein turnover, or extracellular matrix organization [64,65], although such mechanisms were not examined here. Previous study has emphasized BS lectin as a useful marker of glyco-architectural changes in both developmental and pathological contexts [66].

The findings suggest that lectin histochemistry can describe spatial differences in myocardial glycosylation in relation to age and HCM status. Importantly, these results remain associative, derived from post-mortem tissue, and should be viewed as hypothesis-generating rather than mechanistic. The diagnostic potential of lectin-binding patterns has not been evaluated in accessible biological fluids, and their use as biomarkers remains speculative. Although glycosylation changes have been linked to cellular stress, remodelling, and disease in other species [39,43,46], further studies will require validation in serum or blood samples, as demonstrated in other glycomic studies [42,48], along with biochemical confirmation of glycan structures [6,39,47], before clinical application can be considered.

Recent evidence indicate that glycosylation remodelling is a common mechanism in cardiac aging and HCM, with both human and experimental models showing alterations in core and terminal glycan residues [67,68]. In aging hearts, enhanced flux through the hexosamine biosynthetic pathway increases protein O-GlcNAcylation through incorporation of GlcNAc residues, affecting mitochondrial metabolism, calcium handling, and stress signaling, while excessive O-GlcNAc promotes hypertrophy and fibrosis [68,69]. Extracellular matrix glycosaminoglycans also shift, with poly-GlcNAc extensions and α-mannose/α-glucose residues accumulating, thereby stiffening the myocardium and impairing diastolic relaxation [68,70]. Circulating glycome analyses reveal age-related loss of terminal β-D-galactose and sialic acid residues on IgG, enhancing pro-inflammatory Fc activity and linking systemic inflammaging to myocardial remodelling [71,72]. In HCM, proteomic studies show altered abundance of glycoproteins enriched in α-mannose and GlcNAc residues, suggesting receptor and ion-channel glycosylation changes that may exacerbate arrhythmias and hypertrophic signaling [67]. Defective sialylation or loss of terminal β-D-galactose residues disrupts ion-channel trafficking and conduction, while aberrant α-galactosyl and GlcNAc residues on sarcolemmal and matrix proteins impair mechano-transduction and promote fibrosis [73,74]. Collectively, these findings demonstrate that remodelling of both core (α-mannose, α-glucose, GlcNAc) and terminal (sialic acid, β-D-galactose, poly- GlcNAc, α-galactosyl, GlcNAc) residues integrate metabolic, structural, and inflammatory pathways in cardiac aging and HCM [67,71]. This convergence highlights glycosylation not as a passive by-product of metabolic stress, but as an active driver of cardiac phenotype with potential for biomarker discovery and therapeutic intervention [68,69].

With complementary molecular and functional validation, such descriptive lectin profiling could contribute to comparative studies in veterinary and human cardiology [17,39,40,41].

This study has several limitations. First, the lack of complementary molecular approaches (e.g., glycomic profiling or mass spectrometry) prevents definitive identification of the glycans bound by each lectin. Second, although both sexes were represented, sample size did not allow sex-stratified analysis, despite known sex-related variability in HCM. Third, only one region per cardiac chamber was examined; broader mapping could reveal additional glyco-architectural variation. Fourth, the long collection period and case-selection criteria described in Section 3.1 introduced disparities between groups. Finally, as with all post-mortem studies, the possibility for glycan degradation artifacts during tissue processing must be acknowledged [75].

## 5. Conclusions

This study provides the first systematic description of lectin-binding patterns in the feline myocardium across age groups and in the setting of HCM. Using a panel of five plant-derived lectins, we identified chamber-specific and age- or disease-associated differences in cardiac glycan profiles. These findings indicate that myocardial glycosylation in cats is dynamic and influenced by both physiological aging and pathological remodeling. Although the data are descriptive and derived from post-mortem samples, they highlight lectin histochemistry as a useful approach for detecting structural glycan changes in the feline heart. Future work incorporating molecular glycomics, functional analyses, and clinically obtainable specimens will be needed to determine the biochemical drivers of these patterns and to clarify their relevance for disease mechanisms and translational cardiology.

## Figures and Tables

**Table 1:** Group subdivisions into control and HCM categories.

Age Category	Total Cases	Control Cases	HCM Cases
Kitten	13	10	3
Young Adult	23	10	13
Mature Adult	11	7	4
Senior	17	10	7
Total number	64	37	27

**Table 2:** Interpretation for size effect according to Cohen’s guidelines [61].

Size Effect	Mann-Whitney U (r)	Kruskal-Wallis (η^2^):
Low	0.1–0.3	<0.06
Moderate	0.3–0.5	0.06–0.14
Large	>0.5	>0.14

## Data Availability

The data supporting the findings of this study are available from the Department of Veterinary Pathology, University of Agriculture Science and Veterinary Medicine. Data can be accessed upon reasonable request from the authors and with permission from the Department, via the internal repository: Lectin Histochemistry Dataset—Feline Hearts 2025.

## Supplementary Materials

The following supporting information can be downloaded at: https://www.mdpi.com/article/10.3390/life16010020/s1, Document S1: Phyton Script.

## Abbreviations

The following abbreviations are used in this manuscript:

BS	*Griffonia* (*Bandeiraea*) *simplicifolia*
ConA	Concanavalin A
RCA	*Ricinus communis* Agglutinin I
Tomato	*Lycopersicon esculentum* Agglutinin
WGA	Wheat Germ Agglutinin
AT	Atrium
LV	Left Ventricle
RV	Right Ventricle
HCM	Hypertrophic Cardiomyopathy
