# Supplementary material for: Glyco-Architectural Remodelling of the Feline Heart: Age- and HCM-Related Insights from Lectin Histochemistry
Source: Life (Basel). 2025 Dec 22;16(1):20. doi: 10.3390/life16010020 (PMC12843128; doi:10.3390/life16010020)
Supplement: Supplementary file 1 [file life-16-00020-s001.zip › life-3919003-supplementary.pdf]

```python
import os

import tifffile

import numpy as np

import pandas as pd

import tkinter as tk

from tkinter import filedialog, messagebox



def truncate_str(f, n=3):

    s = str(f)

    if '.' in s:

        integer, decimal = s.split('.')

        return float(f"{integer}.{decimal[:n]}")

    return float(s)



def process_lsm_file(file_path):

    rows = []

    try:

        with tifffile.TiffFile(file_path) as tif:

            data = tif.asarray()


        if data.ndim == 3:

            for c in range(data.shape[0]):

                channel_data = data[c]

                stats = {

                    'Filename': os.path.normpath(file_path),

                    'Channel': c,

                    'Min': truncate_str(np.min(channel_data)),

                    'Max': truncate_str(np.max(channel_data)),

                    'Median': truncate_str(np.median(channel_data)),
```

```
                'Std': truncate_str(np.std(channel_data))
            }
            rows.append(stats)

        elif data.ndim == 2:
            stats = {
                'Filename': os.path.normpath(file_path),
                'Channel': 0,
                'Min': truncate_str(np.min(data)),
                'Max': truncate_str(np.max(data)),
                'Median': truncate_str(np.median(data)),
                'Std': truncate_str(np.std(data))
            }
            rows.append(stats)

    except Exception as e:
        print(f"Error processing {file_path}: {e}")

    return rows



def select_folder_and_process():
    folder_selected = filedialog.askdirectory(title="Select Root Folder Containing .lsm Files")
    if not folder_selected:
        return

    all_rows = []
    for root, dirs, files in os.walk(folder_selected):
        for file in files:
            if file.lower().endswith('.lsm'):
                full_path = os.path.join(root, file)
```

```python
            all_rows.extend(process_lsm_file(full_path))

    if not all_rows:
        messagebox.showinfo("Done", "No .lsm files found.")
        return

    df = pd.DataFrame(all_rows)
    output_path = os.path.join(folder_selected, 'lsm_image_stats.xlsx')
    df.to_excel(output_path, index=False)
    messagebox.showinfo("Done", f"Results saved to:\n{output_path}")


# Simple Tkinter GUI
root = tk.Tk()
root.title("LSM Batch Processor")
root.geometry("300x120")

label = tk.Label(root, text="Click the button to select folder and process .lsm files")
label.pack(pady=10)

button = tk.Button(root, text="Select Folder & Start", command=select_folder_and_process)
button.pack(pady=10)

root.mainloop()
```
